# Clotting functional stability of withdrawing blood in storage for acute normovolemic hemodilution: a pilot study

**DOI:** 10.1007/s00540-020-02856-x

**Published:** 2020-09-25

**Authors:** Hirotaka Kinoshita, Junichi Saito, Kishiko Nakai, Satoko Noguchi, Daiki Takekawa, Yoshiko Tamai, Masato Kitayama, Kazuyoshi Hirota

**Affiliations:** 1 Department of Anesthesiology, Hirosaki University Graduate School of Medicine, Zaifu-cho 5, Hirosaki, 036-8562 Japan; 2 Department of Transfusion and Cell Therapy Medicine, Hirosaki University Graduate School of Medicine, Hirosaki, Japan; 3 Division of Operating Center, Hirosaki University Medical Hospital, Hirosaki, Japan

**Keywords:** Clotting function, Acute normovolemic hemodilution, Thromboelastometry, Autologous transfusion

## Abstract

**Purpose:**

This study was conducted to time-course changes of clotting function of withdrawing blood for acute normovolemic hemodilution (ANH).

**Methods:**

Twelve enrolled patients who underwent ANH from August, 2018 to January, 2019. Blood was withdrawn into blood collection pack and shaken at 60–80 rpm for 24 h in room temperature. Clot formation was evaluated using rotational thromboelastometry (ROTEM™) just after blood withdrawal (control) and 4, 8, 12 and 24 h after blood withdrawal. We compared with the control value and each value of extrinsically-activated test with tissue factor (EXTEM), intrinsically-activated test using ellagic acid (INTEM) and fibrin-based extrinsically activated test with tissue factor (FIBTEM).

**Results:**

Maximum clot firmness (MCF) of FIBTEM did not change significantly. MCF of EXTEM was significantly decreased time-dependent manner but all MCF of EXTEM were within a normal range. Maximum percent change in MCF of EXTEM was 12.4% [95% confidence interval (CI): 9.0–15.8%]. The difference in the maximum clot elasticity (MCE) between EXTEM and FIBTEM (MCE_EXTEM_−MCE_FIBTEM_) was significantly decrease from 8 h after blood withdrawal. Maximum percent change in MCE_EXTEM_−MCE_FIBTEM_ was 30.2% (95% CI:17.6–42.9%) at 24 h after blood withdrawal.

**Conclusion:**

Even though the MCE significantly decreased in a time-dependent manner, MCF of FIBTEM and EXTEM was normal up to 24 h storage. The blood of ANH can use for the purpose of hemostasis at least 8 h stored at room temperature after blood withdrawal. Future studies are needed to elucidate the clinical impact on the patient after delayed transfusion of ANH blood with regard to patient’s hemostasis.

**Electronic supplementary material:**

The online version of this article (10.1007/s00540-020-02856-x) contains supplementary material, which is available to authorized users.

## Introduction

Acute normovolemic hemodilution (ANH) is usually carried out to preserve red blood cell, leading reduce the amount of intra- and postoperative allogenic blood transfusion in several clinical settings [1–5]. Moreover, in cardiovascular surgical setting, in addition to the reduction of the risk of postoperative allogeneic blood transfusion, ANH also can reduce the amount of post-operative blood loss [6]. Since ANH blood has not been exposed to the cardiopulmonary bypass (CPB), coagulation factors including fibrinogen and platelet function should be preserved and contribute to hemostasis after surgery.

ANH blood is usually stored at ambient temperature to maintain platelet function during surgery. Storing whole blood at ambient temperature for 24 h has minimal effect on the coagulation activity of plasma, except factor VIII (fVIII) which was loss of 20–30% during first 8 h [7, 8]. However, time dependent changes of clotting function of whole blood has not well determined. We hypothesized that coagulation activity of ANH storing at room temperature would be maintained throughout 24 h. To evaluate our hypothesis, we conducted this study to determine the time dependent changes of the clotting function of the ANH withdrawing blood in storage.

## Methods

The protocol of this prospective observational study was approved by the local Ethics committee, publicized on our hospital homepage (2018–1040) and was registered prior to patient enrollment in a publicly accessible database, the UMIN clinical trial registry, which is one registry of the Japan primary registries network (UMIN000033017, Principal investigator: Junichi SAITO, Date of registration: 15 Jun, 2018). Written informed consent from each patient was waived because blood samples in the tube of blood packs were usually thrown away and the Ethic committee approved the waiver. We enrolled 12 patients who was scheduled to conduct 800 mL of ANH before surgery at Hirosaki University Hospital from August 1, 2018 to January 31, 2019. The primary outcome was the time dependent changes of clotting function of ANH until 24 h after withdrawal of ANH.

### ANH procedure and collected blood sample

The principle indication of ANH in our hospital is an estimated blood loss more than 500 mL or request by surgeons for patients to have more than 10 g/dL of hemoglobin (Hb) after surgery. The amount of blood withdrawal for ANH was 800 mL in all cases, about 20% of blood volume. Autologous whole blood of 400 mL was collected using a decompression blood collecting equipment (Hemo-Quic; AC-181 TERUMO, Tokyo, Japan). After induction of anesthesia in the operating room, blood was withdrawn twice from the central venous line into each standard blood collection pack (JMS Blood Bag CPD400; JMS, Tokyo, Japan) containing citrate phosphate dextrose solution with hemodilution with 500–1000 mL of 6% hydroxyethyl starch (130/0.4) (Voluvein; Fresenius Kabi, Bad Homburg, Germany) to maintain the patient's normovolemia and mean artery pressure ≥ 60 mmHg. ANH procedure took 20–25 min to collect 800 mL of blood. After collection of blood into the blood pack, tube was sealed by tube sealer. Blood in the tube put in the test tube containing citrate phosphate dextrose solution 1.5 mL which was same solution of the standard blood collection packs. As the company supplies the sodium citrate contained tube for measuring rotational thromboelastometry (ROTEM™: Pentapharm GmbH, Munich, Germany), the impact of storage solution on ROTEM™ measurement is considered to be limited. The amount of blood in the tube of each blood pack was 5 mL. About 10 mL of blood sample (mixing the blood of both blood packs) was collected from each patient. ROTEM has some advantages to evaluate the entire clotting function using smaller amount of blood compared with conventional clotting functional test and, thus, ROTEM was suitable to measure clotting function repeatedly in this study. Blood sample in the test tube were shaken 60–80 rpm during 24 h in the room temperature. When a specimen was removed, collected autologous blood was reinfused to the patient.

### Data collection and global tests of hemostasis

**Fig. 1:**
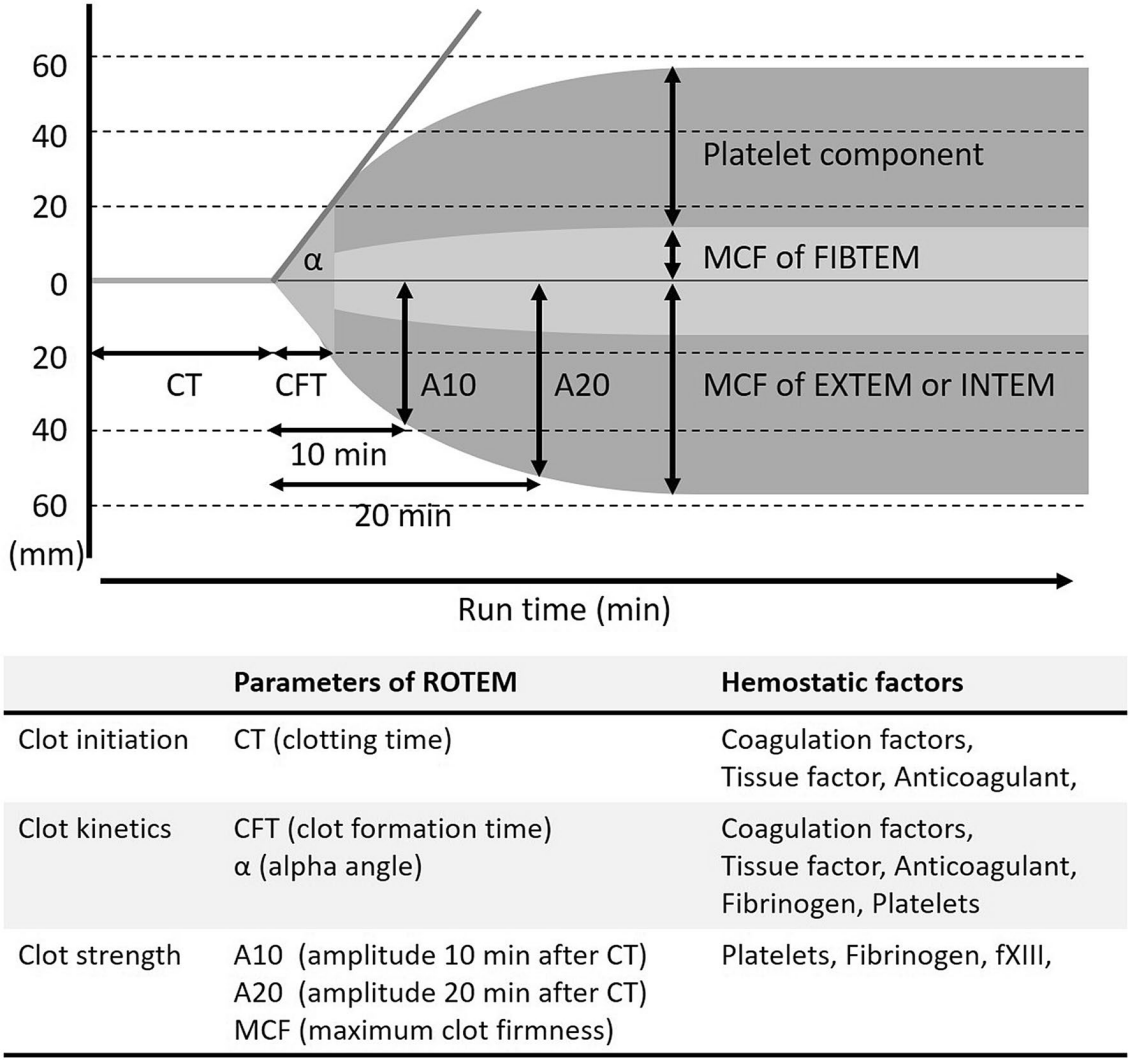
Main parameters of ROTEM™. *EXTEM* extrinsically-activated test with tissue factor, *INTEM* intrinsically-activated test using ellagic acid, *FIBTEM* fibrin-based extrinsically activated test with tissue factor and the platelet inhibitor cytochalasin D

Patient’s demographics, pre-operative platelet counts, prothrombin time and activated partial thromboplastin time were collected. Intraoperative laboratory data, including hemoglobin, hematocrit and platelet counts after withdrawal blood and after re-infusion ANH blood to the patients were also collected. Clot formation was evaluated by ROTEM™ (Fig. 1) and the measurements were at just after withdrawal of blood (within 1 h) and 4, 8, 12 and 24 h after withdrawal. To ensure the accuracy of the ROTEM™ measurement, designated two physicians measured ROTEM™ with the same ROTEM™ machine. The measurements were clotting time (CT), clot formation time (CFT), and maximum clot firmness (MCF) of extrinsically-activated test with tissue factor (EXTEM), intrinsically-activated test using ellagic acid (INTEM) and fibrin-based extrinsically activated test with tissue factor and the platelet inhibitor cytochalasin D (FIBTEM). The platelets contribution to clot elasticity (platelet component parameter) can be calculated from the difference in the maximum clot elasticity (MCE) between EXTEM and FIBTEM (MCE_EXTEM_−MCE_FIBTEM_). MCE was calculated using the following formula: MCE = (MCF × 100)/(100 − MCF) [9].

### Statistical analyses

The statistical analyses were performed using GraphPad Prism V7 (GraphPad Software, La Jolla, CA). Repeated-measures analysis of variance (ANOVA) with Bonferroni post hoc corrections was used to compare with the control value and each value of EXTEM, INTEM and FIBTEM. The sample size was calculated using with G × Power 3.1.9.2 (Universität Düsseldorf, Düsseldorf, Germany). When running a power analysis on a repeated-measures ANOVA with 5 measurements, a power of 0.8, an alpha level of 0.0125, correlation among repeated measures of 0.66, nonsphericity correction of 0.25, and a medium effect size (*f* = 0.25) [10], the required sample size is at least 52. The authors included 12 patients with 60 sample readings. All data are presented as mean ± the standard error of the mean (SEM) or median (25th to 75th percentile). All statistical tests were two-sided, and a *p* value of < 0.05 was considered statistically significant.

## Results

**Fig. 2:**
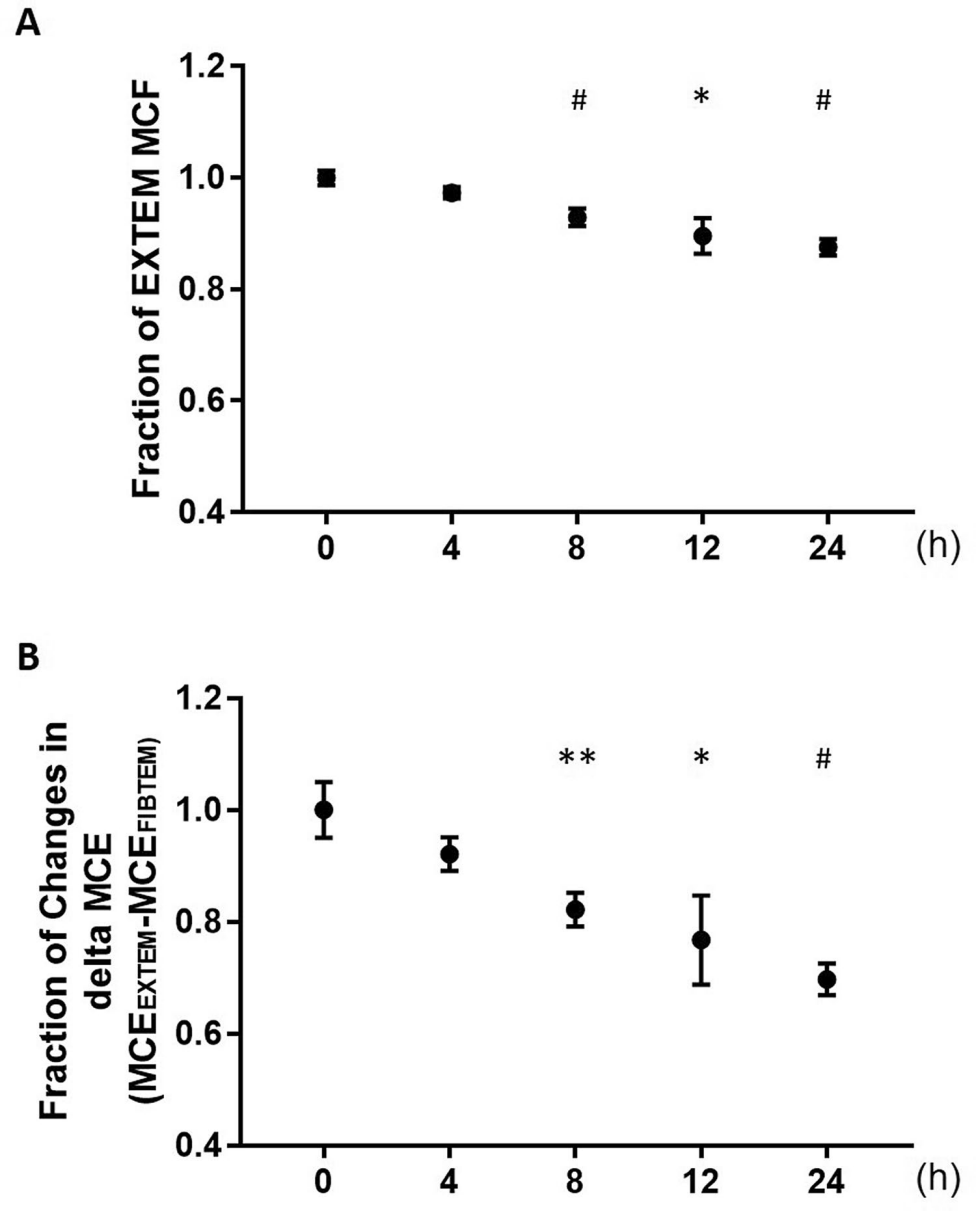
Fraction changes in maximum clot firmness and maximal clot elasticity. **a** Fraction changes in maximum clot firmness of extrinsically-activated test with tissue factor (EXTEM). **b** Fraction changes in the difference in maximal clot elasticity (MCE) between EXTEM and FIBTEM (MCE_EXTEM_−MCE_FIBTEM_); Mean ± SEM, *p < 0.05, **p < 0.01, ^#^*p* < 0.001 vs. 0

Demographic and preoperative laboratory data were presented in the Table 1. The baseline values of each variable of ROTEM™ were within the normal range. CT of EXTEM (Table 2), INTEM (supplemental Table 1) and FIBTEM (Table 3) did not change significantly during the study period. CFT of EXTEM and INTEM was increased in a time-dependent manner but that of EXTEM 24 h after blood withdrawal and INTEM 12 h after blood withdrawal was significantly increased. MCF of FIBTEM did not change significantly but that of EXTEM and INTEM were decreased in a time-dependent manner. That of EXTEM were significantly decreased from 8 h after withdrawing blood but all MCF of EXTEM were within normal physiological ranges. That of INTEM were significantly decreased from 4 h after blood withdrawal and only one reading (49 mm of MCF of INTEM) was below the normal limit (52–72 mm of MCF INTEM) [11]. Maximum percent changes in MCF of EXTEM and INTEM were 12.4% [95% confidence interval (CI): 9.0–15.8%] and 11.6% (95% CI: 6.8–16.4%), respectively (Fig. 2a and Supplemental Fig. 1). MCE of EXTEM and INTEM were significantly decreased time-dependent manner but that of FIBTEM did not changed. MCE_EXTEM_−MCE_FIBTEM_ was significantly decrease from 8 h after blood withdrawal. Maximum percent change in MCE_EXTEM_−MCE_FIBTEM_ was 30.2% [95% CI: 17.6–42.9%] at 24 h after blood withdrawal (Fig. 2b).

**Table 1:** Characteristics of 12 patients and peri-operative laboratory data

Characteristics of patients	
Male, *n* (%)	6 (50)
Age, years	64 (56, 70)
Height, cm	157 (155, 160)
Body weight, kg	57 ± 16

Intra-operative data	
Blood loss, g	604 ± 748
Urine out, mL	1326 ± 1454
Crystalloid solution, mL	2735 ± 1655
Colloid solution, mL	816 ± 489
RBC, *n* (%)	2 (17)
FFP, *n* (%)	2 (17)
PC, *n* (%)	1 (8)

Laboratory data	Pre-ANH	After withdrawal	After re-transfusion
Hb, g/dL	13.9 ± 1.4	9.5 ± 1.6	9.5 ± 1.8
Ht. %	40.9 ± 3.6	27.6 ± 4.2	27.6 ± 5.1
Plt × 10^4^/µL	22.9 ± 5.1	15.8 ± 5.2	14.7 ± 4.4
PT, s	11.5 ± 0.8		
PT-INR	0.99 ± 0.08		
APTT, s	28.5 (27.9, 31.9)		
Fibrinogen, mg/dL	274 (252, 294)		

Mean ± SD, Median (25th, 75th percentile), *n* (%): the number and proportion of patients

*ANH* acute normovolemic hemodilution, *RBC* red blood cells, *FFP* fresh frozen plasma, *PC* platelets concentrate, *Hb* hemoglobin, *Ht* hematocrit, *Plt* platelet count, *PT* prothrombin time, *INR* international normalized ratio, *APTT* activated partial thromboplastin time

**Table 2:** Changes in each variable in EXTEM

EXTEM	Reference range [11]	0	4 h	8 h	12 h	24 h
CT, s	42–74	67 ± 6	55 ± 5	61 ± 7	60 ± 3	64 ± 6
CFT, s	46–148	111 ± 5	121 ± 11	133 ± 9	120 ± 9	141 ± 7**
MCF, mm	49–71	62 ± 1	61 ± 1	58 ± 1***	56 ± 2*	55 ± 1***
MCE, (G dynes/cm^2^)/50	105–235	167 ± 6	155 ± 4	139 ± 6***	131 ± 10*	121 ± 5***

Mean ± SEM

*CT* clotting time *CFT* clot formation time, *MCF* maximum clot firmness, *MCE* maximal clot elasticity;

*p < 0.05, **p < 0.01, ***p < 0.001vs. 0

**Table 3:** Changes in each variable in FIBTEM

FIBTEM	Reference range [11]	0	4 h	8 h	12 h	24 h
CT, s	43–69	59 ± 3	56 ± 5	61 ± 4	47 ± 4	52 ± 3
MCF, mm	9–25	15 ± 2	15 ± 2	13 ± 1	14 ± 1	14 ± 1
MCE, (G dynes/cm^2^)/50	13–27	18 ± 2	19 ± 4	15 ± 2	17 ± 2	16 ± 1

Mean ± SEM

*CT* clotting time, *CFT* clot formation time, *MCF* maximum clot firmness, *MCE* maximal clot elasticity

## Discussion

This prospective observational study revealed clinical important issues. Even though platelet component parameter (MCE_EXTEM_−MCE_FIBTEM_) was significantly decreased in a time-dependent manner from 8 h after blood withdrawal, MCF of FIBTEM remained unchanged and that of EXTEM and INTEM was within reference ranges after 24 h after blood withdrawal. This result suggests that the blood of ANH can use for the purpose of hemostasis at least 8 h stored at the room temperature after blood withdrawal.

Stability of MCF of FIBTEM in this study is supported by the fact that plasma fibrinogen concentration remains unchanged after 24 h after blood collection [7, 8, 12]. In addition, a prospective observational study investigated the coagulation profiles of cold-stored autologous whole blood in using ROTEM™ and revealed that CT of EXTEM and INTEM increased with increasing cold storage duration (7–33 days) but fibrinogen level was not affected by the storage duration. These results indicated that cold-stored autologous whole blood retains fibrin polymerization properties throughout 33 days even though extrinsic and intrinsic coagulation factors decreased in a time-dependent manner [13]. MCF of FIBTEM and plasma fibrinogen concentration have strong correlation in pediatric and adult surgical patients [14, 15]. The unchanged MCF of FIBTEM is estimated to have no change in the plasma fibrinogen concentration in the blood of ANH.

Platelet component parameter (MCE_EXTEM_−MCE_FIBTEM_) was significantly decreased in a time-dependent manner from 8 h after blood withdrawal. Maximum percent change in MCE_EXTEM_−MCE_FIBTEM_ was about 30% at 24 h after blood withdrawal. Even if the whole blood was stored at room temperature and no apparent clot formation in the tube, the platelet function or platelet count could decrease in a time-dependent manner. Transfusion of ANH blood to the patients as soon as possible may be suitable from the perspective of maintaining platelet function or platelet count.

Platelets are recommended to store at room temperature with agitation because refrigeration (2–6 °C) led to rapid clearance from the circulation upon transfusion (t1/2 = 1–2 days), which was significantly reduced compared to room temperature storage (22–24 °C) (t1/2 = 7–9 days) [16]. However, there is growing evidence that cold storage of platelets is superior to room temperature storage of platelets [17]. Platelet rich plasma storing at cold temperature enhances platelet activation and aggregation, and cold storage of platelets in whole blood improves their performance in a panel of functional assays compared to platelets storing at room temperature [18, 19]. Storage temperature of platelets and whole blood is still controversial, and warrant further studies regarding the impact of storage temperature on ANH coagulation.

To our knowledge, the changes in the activity of coagulation factors and platelets on clotting function of whole blood were not well evaluated. However, our present findings support the report that the storage of whole blood at room temperature could change the clotting function of whole blood within several hours [20]. A recent clinical study showed that CFT, α-angle and MCF of EXTEM and FIBTEM significantly changed between withdrawing and reinfusion of the ANH blood after an average of almost 5 h in storage in patients undergoing cardiac surgery [20]. They also showed the decrease in platelets aggregation induced by thrombin receptor activating peptide 6 stimulation during storage, even though the magnitude of change was not significantly correlated with time of storage [20]. These results suggest that platelet function affects the clotting function of ANH and the ROTEM™ measurements.

CFT and MCF of EXTEM and INTEM of the blood of ANH were significantly changed in a time-dependent manner. Activity of fVIII rapidly decreases 20–30% during first 8 h of storage [7, 8]. One retrospective study showed that fVIII was strongly correlated (*r* ≤ − 0.8 or ≥ 0.8) with MCF and CFT of EXTEM and INTEM and MCF of FIBTEM in major surgical patients with hemorrhage [21]. This result suggests that not only platelets, fibrinogen and fXIII [22] but also fVIII had a significant impact on ROTEM™ measurements.

Changes in activity of each coagulation factor during storage at room temperature have been well evaluated in the previous studies [7, 8, 12]. In addition to activity of fVIII, activities of fII, fIX, fX, protein C and protein S were significantly decreased 24 h after blood collection [8]. Even though the activity of all coagulation factors in plasma produced from whole blood stored for 24 h remained above 0.50 U/mL [8], the present results suggested that decreased activity of these factors has an impact on ROTEM™ measurements. Changes in CFT and MCF might reflect of decreased activity of coagulation factors, especially fVIII, in our study.

This study has some limitations. First, the ROTEM™ measurement was once at each measurement point. A coefficient of variation (CV) of ROTEM™ is small; the average ± SD of the CV between-dupilcates vas 4.3% ± 3.8% of CFT and 1.2% ± 1.1% of MCF, respectively [23]. Thus, the impact of ROTEM™ measurement on the result is likely not significant. Second, standard platelet function test and platelet count were not measured in this study. Even though previous studies revealed that the donated blood stored overnight (18–26 h) has little impact on these laboratory examination [24–26], it is well known that many factors could affect platelet function and quality during storage [27], especially oxygen partial pressure, pH and glucose level are affected by the storage duration [26], we should pay more attention to maintain the storage condition. Further studies for the future are needed to elucidate the clinical impact on the patient after delayed transfusion of ANH blood with regard to hemostasis. Third, we did not evaluate the bacterial contamination during storage. Longer duration of storage might increase the risk of bacterial infection. But almost all surgical patients are received anti-bacterial drugs just after induction of anesthesia. The risk of bacterial contamination seems less compared with normal blood donation. Nevertheless, we should pay attention to maintain sterilized condition during ANH procedure and storage.

In conclusion, even though platelet component parameter (MCE_EXTEM_−MCE_FIBTEM_) was significantly decreased time-dependent manner from 8 h after blood withdrawal, MCF of FIBTEM remained unchanged and that of EXTEM was within reference ranges after 24 h after blood withdrawal. This result suggests that the blood of ANH can use for the purpose of hemostasis at least 8 h stored at the room temperature after blood withdrawal. Future prospective studies are needed to elucidate the clinical impact on the patient after delayed transfusion of ANH blood with regard to hemostasis.

## Electronic supplementary material

Below is the link to the electronic supplementary material.

Supplementary file1 (DOCX 34 kb)
