# Supplementary material for: Clotting functional stability of withdrawing blood in storage for acute normovolemic hemodilution: a pilot study
Source: J Anesth. 2020 Sep 25;35(1):35–42. doi: 10.1007/s00540-020-02856-x (PMC7840648; doi:10.1007/s00540-020-02856-x)
Supplement: Supplementary file 1 — Supplementary file1 (DOCX 34 kb) [file 540_2020_2856_MOESM1_ESM.docx]

| Supplemental Table 1. Changes in each variable in INTEM | | | | | | | |
| --- | --- | --- | --- | --- | --- | --- | --- |
| INTEM | Reference range [11] | 0 | 4h | 8h | 12h | 24h |  |
| CT, sec | 137-246 | 170±11 | 172±9 | 167±13 | 176±7 | 175±9 |  |
| CFT, sec | 40-110 | 101±8 | 111±7 | 132±11 | 136±13* | 135±12 |  |
| MCF, mm | 52­-72 | 63±1 | 59±1* | 58±1* | 55±2* | 55±1*** |  |
| MCE,  (G dynes/cm^2^)/50 | 103-250 | 171±9 | 146±5* | 140±7* | 124±8** | 124±5*** |  |
| Mean±SEM;  CT: clotting time; CFT: clot formation time; A10: Amplitude 10min after CT  A20: Amplitude 20min after CT; MCF: maximum clot firmness; MCE: maximal clot elasticity; *p<0.05, **p<0.01, ***p<0.001vs. 0 | | | | | | | |

 Supplemental Figure 1. Fraction changes in maximum clot firmness of intrinsically-activated test using ellagic acid (INTEM)

*

*

#

*

Mean±SEM, *p<0.05, ^#^p<0.001 vs. 0
